# Gender differences in predictors of colorectal cancer screening uptake: a national cross sectional study based on the health belief model

**DOI:** 10.1186/1471-2458-13-677

**Published:** 2013-07-23

**Authors:** Reuben K Wong, Mee Lian Wong, Yiong Huak Chan, Zhu Feng, Chun Tao Wai, Khay Guan Yeoh

**Affiliations:** 1 Division of Gastroenterology and Hepatology, University Medical Cluster, National University Health System, Singapore, Singapore; 2 Saw Swee Hock School of Public Health, National University of Singapore, National University Health System, Singapore, Singapore; 3 Department of Medicine, Yong Loo Lin School of Medicine, National University of Singapore, Singapore, Singapore; 4 Desmond Wai Liver & Gastrointestinal Diseases Centre, Gleneagles Medical Center, Singapore, Singapore

**Keywords:** Colorectal cancer, Screening, Health belief model, Gender differences

## Abstract

**Background:**

Colorectal Cancer (CRC) is rapidly rising in Asia, but screening uptake remains poor. Although studies have reported gender differences in screening rates, there have been few studies assessing gender specific perceptions and barriers towards CRC screening, based on behavioral frameworks. We applied the Health Belief Model to identify gender-specific predictors of CRC screening in an Asian population.

**Methods:**

A nationwide representative household survey was conducted on 2000 subjects aged 50 years and above in Singapore from 2007 to 2008. Screening behaviour, knowledge and beliefs on CRC screening were assessed by face-to-face structured interviews. The response rate was 88.2%.

**Results:**

26.7 percent had undergone current CRC screening with no gender difference in rates. Almost all agreed that CRC would lead to suffering (89.8%), death (84.6%) and would pose significant treatment cost and expense (83.1%). The majority (88.5%) agreed that screening aids early detection and cure but only 35.4% felt susceptible to CRC. Nearly three-quarters (74.3%) of the respondents recalled reading or hearing information on CRC in the print or broadcast media. However, only 22.6% were advised by their physicians to undergo screening. Significantly more women than men had feared a positive diagnosis, held embarrassment, pain and risk concerns about colonoscopy and had friends and family members who encouraged screening. On multivariate analysis, screening uptake showed a positive association with worry about contracting CRC and a physician’s recommendation and a negative association with perceived pain about colonoscopy for both genders. For women only, screening was positively associated with having attended a public talk on CRC and having a family member with CRC, and was negatively associated with Malay race and perceived danger of colonoscopy.

**Conclusions:**

CRC screening remains poor despite high levels of awareness of its benefits in this Asian population. Race, worry about contracting cancer, psychological barriers, and cues from the doctor and a public talk on CRC were associated with screening with gender specific differences. Strategies to increase CRC screening uptake should consider gender specific approaches to address psychological barriers and increase disease susceptibility through public health education and active promotion by physicians.

## Background

Colorectal Cancer (CRC) is the commonest cancer among men, and the second commonest among females in Singapore. For the period 2002 to 2006, the age-standardized rates (ASR) for CRC were 40.2 per 100,000 per year for men and 28.8 per 100,000 per year for women [1]. This average population risk for developing colorectal cancer is equal to, if not greater than many Western countries [2].

If detected early, CRC is curable. Prospective trials have demonstrated significant mortality reduction with early detection of cancer and adenomas (the precursor lesions of colorectal cancer) [3]. Established screening modalities for CRC and adenomas exist–namely fecal occult blood testing (FOBT), endoscopy (colonoscopy and sigmoidoscopy) and radiologic imaging (barium enema and Computerized Tomographic (CT) colonography) [3]. There is a shift away from recommending the use of the barium enema as a screening tool due to its lower sensitivity and specificity. The latter is a back-up screening option if optical colonoscopy fails, and CT colonography is not available [2-5]. In Singapore, although CT colonography is now within the local CRC screening guidelines [4], FOBT and endoscopy remain the 2 modalities that are most widely available to healthcare providers. In Asia, national guidelines for CRC screening are currently available in Singapore, Japan, Korea and Taiwan [4-8]. This means that most countries in Southeast Asia do not have screening guidelines or coordinated screening programs in place. Currently, free screening is only available in Taiwan through a national health insurance program [8]. In Singapore, national CRC screening guidelines were introduced in 2010, where screening is recommended for an average-risk individual beginning at age 50, and earlier for higher risk individuals [4]. The local screening algorithm and recommended modalities are derived from the American Gastroenterological Institute, World Gastroenterological Organization and Asia Pacific Consensus Recommendations/Guidelines for Colorectal Cancer Screening [2-5]. Screening is offered by both public and private healthcare providers, but it is not mandated and is only available on a co-payment basis. Individuals are also not constrained to screening by one modality. For example, an individual may have gone for a FOBT followed by colonoscopy, if indicated; or alternatively may have opted for an endoscopy directly.

Despite good evidence supporting CRC screening, studies estimate that only half of the eligible population in the United States have been screened, and rates in Asia are believed to be even lower [9]. A large comprehensive telephone survey in Hong Kong which applied a behavioral model to assess obstacles to CRC screening found only a tenth of respondents had been screened. However, the survey included individuals below the recommended screening age, and the findings did not describe “currency with screening” as per the guidelines [10]. In addition, the study did not examine gender differences in screening. Many reasons for the low screening uptake have been postulated, among which are poor knowledge about CRC, and the lack of awareness of screening benefits and early detection [11]. In Singapore, a telephone survey conducted more than ten years ago of patients from a surgical practice database reported the lack of knowledge and awareness of CRC as major issues [12].

Studies have reported gender differences in screening rates, suggesting the need for a gender specific approach to promote CRC screening. However, there have been few publications describing gender specific perceptions and barriers of the screening eligible population towards CRC screening, based on behavioral frameworks such as the Heath Belief Model (HBM). McQueen et al. examined the role of perceived susceptibility on CRC screening behavior in Caucasian males, but the study was based on intention to screen, rather than the respondents actually having undergone screening [13]. Focus groups have explored barriers, attitudes and preferences by gender [14-16], but the magnitude of the factors from these qualitative research studies is unclear. Therefore, large quantitative studies are needed to determine the extent of behavioral factors influencing the uptake of CRC screening by gender. The findings will guide future interventions on whether there is a need for a more gender-specific approach to promote CRC screening.

The HBM is a “value-expectancy” model developed to explain an individual’s health actions under conditions of uncertainty and consists of 5 components-perceived susceptibility, severity, benefits, barriers and cues that contribute to an individual’s likelihood of taking action [17]. It stands out among socio-psychological models of health-related screening behavior as most frequently cited and researched.

This study was conducted to determine the prevalence of uptake of colorectal cancer screening and knowledge about CRC among adults aged 50 years or more in the general population in Singapore. In addition, we applied the HBM to compare gender differences in the factors associated with CRC screening.

## Methods

We conducted a nationwide survey from 2007 to 2008 on a proportional stratified random sample of 2,000 household units, obtained from a sampling frame of all households in Singapore. The sample size was computed to be 1629, to yield a 2% error of margin at the 95% confidence interval (CI) level, for an estimated CRC screening rate of 22%. No local data exists on screening rates, so we decided to take the lower range of prevalence studies done overseas on “screening rates among eligible individuals” [3,18]. After accounting for an estimated non-response rate of 20%, the final sample size was estimated to be 2000 subjects. A proportional stratified random sample of 2000 dwelling units was selected from a sampling frame of all households stratified by housing type in Singapore. Eligible subjects were defined as any individual within the selected defined dwelling who was 50 years of age and above [3]. If there were more than one eligible subject in the selected household, only one subject was randomly selected using the Kish Grid method to participate in the survey. Excluded were subjects who were unable to provide coherent answers.

**Figure 1:**
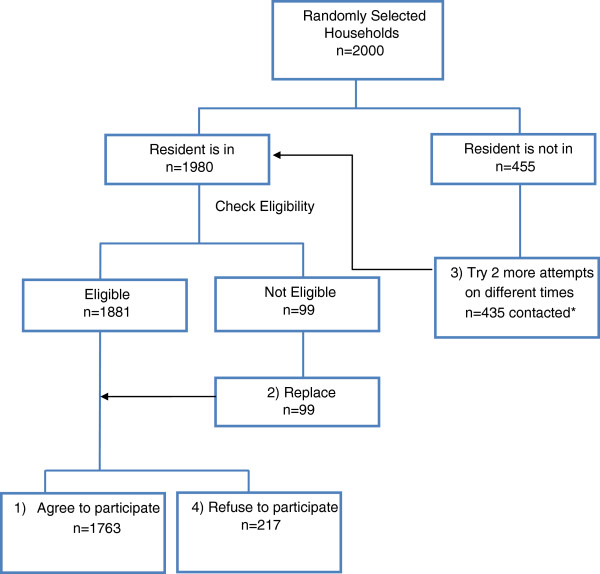
Survey Process Workflow. *The 20 non-contactables were assumed to be eligibles. Hence the response rate is 1763/2000 ie 88.2%.

The survey was conducted by 40 interviewers who underwent formal training. This ensured a standardized interview technique to reduce interviewer and interviewee bias. Interviewers were assigned a list of addresses within a district and went door-to-door conducting the interviews. The survey was conducted using face-to-face interviews with a structured questionnaire. There were 4 possible scenarios to each interview encounter (Figure 1): 1) resident was in and eligible for the study–the interview was conducted. 2) resident was in but not eligible because he was under the age of 50 years–the interviewer would then move to the next-door dwelling on the right in the same block till an eligible resident was found. This ensured that the non-eligible person would be replaced within the same housing type to reduce selection bias. Dwellings in the same block are similar in type with regard to size and number of rooms; hence, residents living in the same block are very similar in socio economic status. 3) no response because nobody was in–the interviewers would then try again for two separate attempts at different times. 4) refusal–the interviewers recorded this as a non-respondent and then proceeded to the next address on the list.

The study was approved by the National Healthcare Group Domain Specific Review Board, Singapore. Informed consent was obtained from the subjects orally, using a set template read-out by the interviewers, without needing a signed consent document.

### Questionnaire

Data were collected on socio-demographic characteristics such as age, gender, race, marital status, education and income. The questionnaire also included screening questions on a personal history of CRC, colorectal adenomas or inflammatory bowel disease. These subjects were at higher risk of CRC, and would not be considered screening candidates. We also asked about knowledge specifically on prevalence, awareness of endoscopic screening and the common presenting symptoms of CRC. The response options for knowledge statements were “Yes, No or Don’t Know”.

The main dependent variable was currency with screening. We followed the American Gastroenterological Association CRC guidelines and defined it as a FOBT within the last 1 year, sigmoidoscopy within the last 5 years or colonoscopy within the last 10 years [3].

Questions covering the 5 domains of the HBM included perceived susceptibility to acquiring CRC, perceived severity of the disease, benefit of going for screening, barriers to action and cues to action. For example, perceived susceptibility was assessed by 4 items-chance of developing CRC, worry about getting CRC, whether it is fated to get CRC and whether one can prevent oneself from getting it. Barriers were assessed by 7 items and these included the fear, unwillingness to find out the results, perceived danger, pain, embarrassment, financial cost and inconvenience of screening.

The response options for these HBM items were “disagree”, “agree” and “unsure”. In the analysis, all the domains except barriers were collapsed into binary measures as follows (i) agree and (ii) disagree and unsure combined. The proportions who agreed were presented in the tables. For barriers, the response categories were also collapsed into binary measures, but with the following difference (i) disagree and (ii) agree and unsure combined. The proportion who disagreed rather than those who agreed was presented for the following reasons: First, barriers were found by research to be the most powerful single predictor of inaction, and the response to barriers was less affirmative [19]. Second, research on health behavior and attitudes showed that some respondents were particularly likely to agree when questions were put in the agree-disagree format [20].

Cronbach alphas to assess internal consistency of the items within each domain were computed. Except for the domain of perceived severity which had a Cronbach alpha of 0.78, Cronbach alphas for the other domains ranged from 0.33 to 0.64 and were below the acceptable values of 0.70. Because of the weak Cronbach alphas, we decided to analyze each item separately. This also allowed us to target specific interventions to address the statistically significant items within each domain.

The survey instrument was piloted on 10 subjects (relatives of in-patients visiting in the wards), who also provided feedback on the questions posed. This resulted in questions being re-phrased to ensure clarity, and the interviewers being briefed on how to phrase the items, so as to reduce ambiguity. To further reduce interviewee’s bias, cough and sore throat were inserted as “red-herring” symptoms to screen out individuals who were mechanically answering all the responses in a unitary unthinking manner. However a subgroup analysis of the individuals who got these 2 questions wrong showed this not to be the case.

### Data analysis

All analyses were stratified by gender. Chi-square test was used to compare differences in knowledge of possible CRC symptoms and constructs in the HBM between genders. Predictive factors using the constructs from the Health Belief Model, for CRC screening behavior were determined using multivariate logistic regression adjusted for demographics (race, religion, occupation, income, education, marital status, and housing), family history for colorectal adenoma and all other variables in the HBM. All the adjusted odds ratios with 95% CI in the multivariate model were presented. A two-tailed p-value of p < 0.05 was considered to be statistically significant. Statistical analyses utilized SPSS software system (version 17.0 for Windows, SPSS Inc., Chicago, IL).

## Results

### Surveyed population

A total of 1,763 out of 2000 eligible subjects responded to the survey, giving a 88.2% response rate. 20 respondents had to be excluded because they had pre-existing CRC or Inflammatory Bowel Disease. The final data set consisted of 1743 subjects (1050 women and 693 men). The mean age of the respondents was 61.3 years with 60.2% females.

Table 1 compares the socio-demographic characteristics of the male and female respondents. There was a significantly higher proportion of working men as compared with women (46.1% vs. 20.3%, p < 0.001). Similarly, a disparity existed in educational level, with only 9.7% of women compared with 19.7% of men having received a tertiary education and 21.5% of women versus 7.8% of men having no formal schooling. The distribution by race, age, dwelling type racial and educational level was similar to that in the general population for this age group (data not shown) [21].

**Table 1:** **Socio**-**demographic characteristics of the surveyed sample**, **Singapore**, **2007**-**2008**

**Characteristics**	Men (%)*		Women (%)*		**P value**
	**n = 693**		**n = 1050**		
	**n**	**(%)**	**n**	**(%)**	
**Mean age in years **(**range**)	62.1	(50–91)	61.2	(50–93)	
**Race**					
Chinese	548	(79.1)	862	(82.0)	0.250
Indian	67	(9.6)	90	(8.6)	
Malay	64	(9.3)	72	(6.9)	
Others^#^	14	(2.0)	26	(2.4)	
**Religion**					
Buddhism	259	(37.4)	429	(40.9)	0.003
Christianity	104	(15.0)	190	(18.1)	
Taoism	67	(9.7)	120	(11.4)	
Islam	77	(11.1)	78	(7.5)	
Hinduism	46	(6.6)	75	(7.1)	
No Religion	140	(20.2)	158	(15.0)	
Occupation*					
Retired	281	(40.5)	293	(27.9)	<0.001
Working	319	(46.1)	213	(20.3)	
Homemaker	4	(0.6)	416	(39.6)	
Unemployed	89	(12.8)	128	(12.2)	
**Years of schooling**					
Nil	54	(7.8)	226	(21.5)	<0.001
1-6	232	(33.5)	379	(36.1)	
7-12	270	(38.9)	342	(32.6)	
>12 (tertiary)	137	(19.7)	102	(9.7)	
**Dwelling type**					
HDB 1–3 room	193	(27.9)	290	(27.6)	0.660
HDB 4–5 room	406	(58.6)	634	(60.4)	
Private condominium	94	(13.6)	126	(12.0)	
**Marital Status**					
Married	616	(88.9)	879	(83.7)	<0.001
Single	46	(6.7)	60	(5.7)	
Widowed	17	(2.4)	95	(9.0)	
Divorced	14	(2.0)	16	(1.5)	

*Excludes missing values.

^# ^Europeans/Eurasians.

### CRC screening behavior

**Figure 2:**
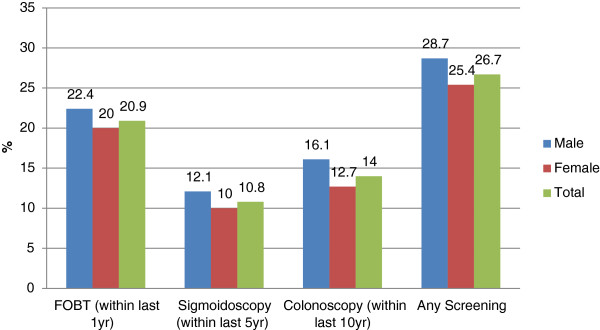
CRC Screening uptake among males and females.

The overall currency with CRC screening rate was 26.7%, with no significant difference between males and females (28.7% vs. 25.4%, p = 0.12). As shown in Figure 2, FOBT was the most commonly utilized screening modality (20.9%), followed by colonoscopy (14%). Females were less likely to have undergone a colonoscopy for CRC screening (OR = 0.76, 95%CI 0.58–0.99, p = 0.041).

### Knowledge on CRC

**Figure 3:**
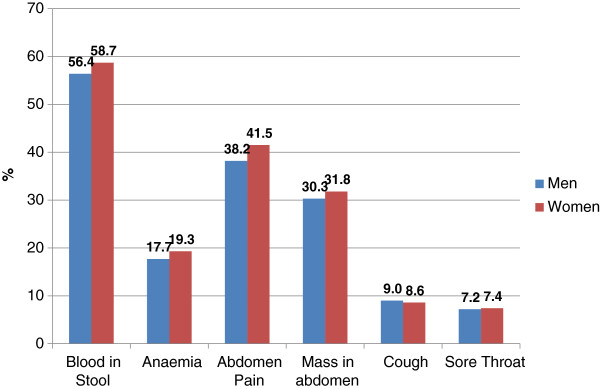
Knowledge on presenting symptoms of colorectal cancer.

Regarding awareness on the prevalence of cancers, 64% of men and 66% of women recognized that CRC was among the top 3 cancers locally. When asked about their awareness on endoscopy as a screening modality, 54% of males and 55% of females were aware that endoscopy was “one way to check for colon cancer”. Figure 3 shows the knowledge of the respondents on possible symptoms of CRC. At least half of the respondents could identify blood in the stool as a possible symptom of CRC, less than 10% wrongly said cough and sore throat were CRC symptoms. There were no significant differences between males and females in their responses.

### The health belief model

Table 2 compares the 5 domains of the HBM by gender, Slightly more than a third of subjects (35.4%) believed that they had some or high chance of developing CRC, with significantly more men than women (39.2% vs. 32.5%, p = 0.04) holding this belief. Nearly half were resigned to the fact that fate determined if they would get the disease. Nearly 65% believed they could do something to prevent themselves from acquiring CRC. On the perceived severity of the disease, there was clear awareness of the impact of CRC morbidity on all domains of life with no gender differences. Almost all agreed that CRC would lead to suffering (89.8%), death (84.6%) and would pose significant treatment cost and expense (83.1%). Similarly, the majority (88.5%) agreed that early screening would detect the cancer at a sufficiently early stage to be cured.

**Table 2:** Prevalence of domains in the health belief model by gender

**Statement**	Men (% agree)*		Women (% agree)*		**P value**
	**n = 693**		**n = 1050**		
	**n**	**(%)**	**n**	**(%)**	
**Susceptibility**					
I have some/high chance of developing CRC	272	(39.2)	341	(32.5)	0.004
I never worry about getting CRC	199	(28.7)	319	(30.4)	0.45
It is fated that I will get CRC	301	(43.4)	484	(46.1)	0.26
I can prevent myself from getting CRC	463	(66.8)	653	(62.2)	0.049
**Severity**					
CRC leads to suffering	629	(90.7)	937	(89.2)	0.34
CRC leads to death	597	(86.2)	878	(83.6)	0.16
CRC affects my family	580	(83.6)	895	(85.2)	0.38
CRC affects my social life	595	(85.9)	890	(84.8)	0.54
CRC is expensive to treat	563	(81.3)	885	(84.3)	0.11
**Benefit**					
CRC screening helps detect cancer early	624	(90.0)	918	(87.4)	0.096
**Cues**					
I have attended a public talk on CRC	49	(7.0)	101	(9.6)	0.068
I heard/read about CRC from TV/newspapers	499	(72.0)	793	(75.5)	0.11
I heard about CRC from friends or relatives	269	(38.8)	450	(42.9)	0.094
Doctor recommended CRC screening to me	139	(20.1)	256	(24.4)	0.037
Friends told me to go for CRC screening	80	(11.6)	187	(17.8)	<0.001
My family told me to go for CRC screening*	106	(15.3)	226	(21.5)	0.001
Family history of CRC	38	(5.5)	64	(6.1)	0.238
**Barriers**	**Men (% disagree)**		**Women (% disagree)**		**p-value**
	**n**	**(%)**	**n**	**(%)**	
I rather not know if I had CRC	420	(60.6)	606	(57.7)	0.23
I am afraid of finding out if I have CRC	403	(58.2)	487	(46.4)	<0.001
Screening is expensive	123	(17.8)	167	(15.9)	0.30
Colonoscopy is dangerous	396	(57.2)	540	(51.4)	0.017
Colonoscopy is painful	236	(34.0)	273	(26.0)	<0.001
Colonoscopy is embarrassing	453	(65.3)	391	(56.4)	<0.001
Inconvenient to see doctor for CRC screening	417	(60.2)	562	(53.5)	0.006

*Excludes missing values.

Among the potential barriers to CRC screening, the unwillingness and fear of finding out that they had cancer were reported by more than 40% of respondents. Women were significantly more likely to have fears about discovering they had the disease than men (53.6% vs. 41.8%, p < 0.001). Most of the respondents (83.4%) were concerned about cost of the screening test. Focusing on colonoscopy as a screening modality, the pain, embarrassment and perception of the procedure as dangerous were cited by a significantly higher proportion of females than males. More than half of the respondents disagreed that inconvenience in seeing a physician was a barrier to CRC screening.

Finally, when cues to action were assessed, nearly three-quarters of the respondents recalled having read or heard about CRC in the print or broadcast media. A low 22.6% had been encouraged to go for screening by their doctor, a percentage only slightly higher than prompting from friends and family members. Women were significantly more likely than men to report encouragement from their doctor, family, and friends to go for CRC screening.

### Factors in the HBM which predicted screening behavior

Tables 3 and 4 compare the bivariate analysis of demographic characteristics and HBM factors associated with CRC screening between men and women. CRC screening was significantly associated with race among women but not men. CRC screening increased significantly with educational level and showed a borderline significant association with older age among men but not women. On examining the HBM domains (Table 4), CRC screening for both genders showed significant associations with worry about getting CRC, all items in the barriers domain such as fear of finding out about having CRC, unwillingness to know about CRC, perception that colonoscopy was dangerous, painful and embarrassing, cost and the inconvenience of seeing the doctor and some items in the ‘cues’ domain such as recommendations by the doctor, family and friends. For women only, CRC screening showed significant associations with the belief that CRC screening detects cancer in its early stage, having attended a public talk and having read or heard about CRC in the print or broadcast media in the past year, and a family history of CRC. A finding among men only was the significant association of CRC with the belief that one could prevent oneself from getting CRC.

**Table 3:** **Percentage distribution of colorectal cancer screening by socio**-**demographic characteristics among men and women in Singapore**

**Statement**	**Men**		**P value**	**Women**		**P value**
	(Screened for CRC)*			(Screened for CRC)*		
	**n = 693**			**n = 1050**		
	**n**	**(%)**		**n**	**(%)**	
**Race**						
Chinese, Indians, others^≠^	186	(29.2)	0.295	263	(26.2)	0.027
Malays	15	(23.1)		11	(14.7)	
**Age group** (**years**)						
50-59	84	(27.9)	0.076	123	(24.2)	0.973
60-69	56	(26.2)		101	(28.5)	
70-79	35	(28.9)		41	(25.5)	
> = 80	21	(47.7)		8	(18.2)	
**Years of schooling**						
Nil	12	(21.8)	0.017	52	(23.0)	0.111
1-6	60	(25.9)		86	(22.6)	
7-12	73	(27.0)		93	(27.2)	
>12 (Tertiary)	51	(37.5)		30	(29.7)	
**Dwelling type**						
HDB 1–3 room	43	(22.3)	0.037	55	(19.0)	0.013
HDB 4–5 room	120	(29.6)		168	(26.5)	
Private condominium	32	(34.0)		39	(31.0)	

^≠^The majority >85% are Chinese in the group. * Excludes missing values.

**Table 4:** Percentage distribution of colorectal cancer screening by domains of health belief model among men and women in Singapore

**Statement**	**Men**		**P value**	**Women**		**P value**
	(Screened for CRC)*			(Screened for CRC)*		
	**n = 693**			**n = 1050**		
	**n**			**(%)**	**n**	**(%)**	
**Susceptibility**						
*Worry sometimes*/*always about getting CRC*						
Yes	71	(35.3)	0.014	103	(31.4)	0.002
No	130	(26.0)		170	(22.7)	
*Some or high chance of developing CRC*						
Yes	83	(30.2)	0.478	98	(28.0)	0.161
No	118	(27.7)		175	(24.0)	
*Fated I will get CRC*						
Yes	129	(26.0)	0.659	79	(26.0)	0.169
No	144	(24.8)		122	(30.7)	
*Can prevent myself from getting CRC*						
Yes	152	(32.5)	0.002	171	(25.5)	0.848
No	49	(21.0)		102	(25.0)	
**Seriousness**						
*CRC leads to suffering*						
Agree	186	(29.2)	0.295	237	(24.6)	0.134
Disagree	15	(23.1)		36	(31.0)	
*CRC leads to death*						
Agree	176	(29.1)	0.496	219	(24.3)	0.083
Disagree	25	(25.8)		54	(30.5)	
*CRC affects my family*						
Agree	176	(30.0)	0.072	235	(25.6)	0.620
Disagree	25	(21.7)		38	(23.8)	
*CRC affects my social life*						
Agree	180	(29.9)	0.076	229	(25.1)	0.630
Disagree	21	(21.2)		44	(26.8)	
*CRC is expensive to treat*						
Agree	161	(28.2)	0.601	228	(25.1)	0.672
Disagree	40	(30.5)		45	(26.6)	
**Benefit**						
*CRC screening helps detect cancer early*						
Yes	186	(29.5)	0.158	249	(26.4)	0.028
No	15	(21.4)		24	(17.6)	
**Cues**						
*Attended public talk on CRC*						
Yes	17	(34.7)	0.334	40	(38.8)	0.001
No	184	(28.2)		233	(23.9)	
*Heard about CRC from TV*/*newspapers*						
Yes	156	(30.9)	0.632	218	(26.8)	0.001
No	45	(28.7)		55	(20.8)	
*Heard of friends*/*relatives with CRC*						
Yes	88	(32.4)	0.086	139	(30.1)	0.002
No	113	(26.3)		134	(21.8)	
*Doctor recommended CRC screening to me*						
Yes	78	(53.3)	<0.001	109	(41.4)	<0.001
No	123	(22.0)		164	(20.1)	
*Friends told me to go for CRC screening*						
Yes	38	(46.9)	<0.001	69	(35.9)	<0.001
No	163	(26.3)		204	(23.0)	
*Family told me to go for CRC screening*						
Yes	47	(43.9)	<0.001	80	(34.5)	<0.001
No	154	(25.9)		193	(22.8)	
*Family history of colorectal cancer*						
Yes	10	(27.8)	0.899	31	(44.3)	0.004
No	191	(28.8)		243	(24.1)	
**Barriers**						
*I*’*m afraid to find out if I have CRC*						
Disagree	133	(32.6)	0.007	145	(29.0)	0.01
Agree	68	(23.2)		128	(22.1)	
*I*’*d rather not know if I had CRC*						
Disagree	137	(32.2)	0.01	173	(27.8)	0.028
Agree	64	(23.2)		100	(21.9)	
*Colonoscopy is dangerous*						
Disagree	136	(33.9)	<0.001	174	(31.4)	<0.001
Agree	65	(21.7)		99	(18.9)	
*Colonoscopy is painful*						
Disagree	106	(44.5)	<0.001	106	(37.9)	<0.001
Agree	95	(20.5)		167	(20.9)	
*Colonoscopy is embarrassing*						
Disagree	154	(33.6)	<0.001	175	(28.8)	<0.001
Agree	47	(19.3)		98	(20.9)	
*Screening is expensive*						
Disagree	49	(39.2)	0.004	62	(36.3)	<0.001
Agree	152	(26.4)		211	(23.3)	
*Inconvenient to see doctor for CRC screening*						
Disagree	135	(32.0)	0.017	163	(28.2)	0.01
Agree	66	(23.7)		110	(22.0)	

*Excludes missing values.

Table 5 shows the adjusted odds ratios of CRC screening in the multivariate logistic regression analysis. When all potential explanatory variables were included in the final model, the following variables remained significantly associated with CRC screening. Malay females were half as likely as non-Malay females to participate in CRC screening. Females who perceived that colonoscopy was a dangerous procedure were significantly less likely to go for CRC screening while those who had attended a public talk and had a family history of CRC were significantly more likely to go for CRC screening. For both genders, worry about getting CRC and recommendation by a doctor to go for screening were positively associated with screening while the perception about colonoscopy as a painful procedure showed a negative association with screening.

**Table 5:** Multivariate logistic regression model indicating adjusted odds ratios for colorectal screening among men and women in Singapore

	Men *		Women*	
	**n = 693**		**n = 1050**	
	**Adjusted# OR**	**95% CI**	**Adjusted# OR**	**95% CI**
Malay				
No+	Ref		Ref.	
Yes	0.79	0.50-1.25	0.45	0.22-0.91
Age	1.02	1.00-1.04	1.01	0.98-1.02
Educational level	1.14	1.03-1.26	0.97	0.91-1.23
Worry about getting CRC				
No	Ref.		Ref.	
Yes	1.57	1.06-2.30	1.44	1.06-1.96
Colonoscopy is potentially dangerous				
No	Ref.		Ref.	
Yes	0.98	0.66-1.57	0.64	0.46-0.87
Colonoscopy is painful				
No	Ref.		Ref.	
Yes	0.38	0.26-0.55	0.59	0.43-0.83
Attended a public talk on CRC				
No	Ref		Ref.	
Yes	1.23	0.40-1.65	1.70	1.08-2.67
My personal doctor had recommended CR screening				
No	Ref.		Ref.	
Yes	3.50	2.33-5.27	2.35	1.71-3.22
Family history of CRC				
No	Ref.		Ref.	
Yes	1.05	0.65-1.70	2.50	1.49-4.19

*Excludes missing values.

# Adjusted for race, age, education, dwelling type, and all variables in HBM.

Ref-referent group.

+The majority >85% are Chinese in the group.

## Discussion

CRC screening had been proven to reduce morbidity and mortality from the disease, but only 26.7% of our surveyed population had a current CRC test. This rate is low compared to currency with screening rates reported in the United States [9], but comparable to a population-based study in Ontario, Canada [22]. Like our study, the Canadian study was conducted in a relatively urban community with access to publicly funded healthcare and in a population with a similarly high incidence of CRC [22]. However, our screening rates are unacceptably low, when 73.3% of the eligible subjects had not gone for screening, despite the fact that nearly 90% were aware that screening helps detect CRC at an early stage which could be cured. There was clearly a discordance between knowledge and action, which we sought to explain using the HBM.

In our study, we found important differences in the HBM domains between men and women that would support a gender specific approach to promoting CRC screening. Firstly, women had more concerns about the risks that might arise from an endoscopic examination, mirroring similar concerns from their Western female counterparts [23]. In addition, they also had greater fears about receiving a positive diagnosis of CRC. This suggests a need for a personalized approach for females, where these intimate issues can be discussed on a one-to-one basis, and reassurance and support offered accordingly. Second, substantially more women cited having family or friends who encouraged them to go for screening, but this surprisingly did not have a significant effect on improving actual screening behavior. This suggests tapping on the network of family and friends will be potentially a useful method in getting across awareness of CRC screening, but unto itself may still be inadequate in actually changing screening behavior.

It was interesting to note that Malay females were significantly less likely to have gone for screening as compared to their Chinese, Indian and European/Eurasian counterparts. This difference persisted despite controlling for economic and educational factors, and the similarity in healthcare access ethnic groups. This ethnic difference in screening behavior was not seen in men, and we would posit there were gender-specific socio-cultural factors that could have influenced Malay women to lag behind in CRC screening. Further studies are needed to elucidate these factors.

The finding that only a small proportion of patients have been encouraged by their doctors to undergo screening was worrying. This was in contrast to a study amongst Medicare consumers in the United States, where 72% had received a doctor’s recommendation to consider CRC screening [24]. More should be done to address this issue because our study found that a doctor’s recommendation for screening was a strong predictor of positive screening behavior in both men and women (adjusted OR 3.50 and 2.35 respectively). This mirrored findings from a recent British study, suggesting that 84% of respondents not only wanted information on the risks and benefits of screening, but also sought recommendation from an “authoritative” source [25]. At the time of the survey, there was no national drive to promote CRC screening. An education program and revised set of screening guidelines directed at doctors have just been launched. It will be beneficial to repeat the survey in a few years’ time, and review what effect these interventions have had practically on the doctors’ behavior and on screening uptake.

Our finding on the positive association of attendance at public talks with CRC screening among women suggests that public education and the media have been effective channels in raising awareness about CRC. The low level of self-perceived susceptibility (39.2% in men and 32.5% in women) is a concern. This was not dissimilar to rates in a large pan-European study, where only 31% of the respondents believed they were at risk of contracting CRC [26]. Outreach to the public through educational programs, advertisements and the mass media are a cost-effective way of increasing awareness of CRC and its rapidly rising incidence in the Asian population. A more impactful method may be through personal encounters. Among women, having a family member with CRC was a strong positive predictor of screening behavior, and one postulate was that seeing or hearing about a relative contacting a malignancy was a reminder of one’s susceptibility and a cue for screening. Among men no such association was found, which would suggest that such a targeted approach pivoted on a family member with the disease would be less impactful than in women.

Our study had some limitations. We did not distinguish between subjects who might have gone for CRC testing for diagnostic purposes rather that for screening. However, we attempted to reduce this bias by excluding respondents who had significant colonic pathology from the analysis on the factors associated with screening. We recognized this was a cross-sectional study, and hence could not exclude temporal bias in the causal effect relationship of psychosocial beliefs and attitudes on CRC screening. For example, the perception of pain could have occurred after CRC screening rather than perceived pain preceding screening. Another limitation was that our study was based on self-reporting, hence some respondents might over-report socially desirable attitudes. We attempted to reduce this bias by training our interviewers to ask questions in an objective and reassuring manner.

However, our study has several strengths. This is the first large scale study describing gender differences in the various behavioral components of the HBM, in an Asian country with a high CRC incidence. There had been a study describing barriers to CRC screening using the HBM, but gender differences were not reported [10]. In addition, our survey was conducted on a moderately large nationally representative sample of a CRC screening eligible population. Moreover, the survey has yielded a high response rate. This study is also one of the few to describe a more clinically relevant “currency with screening” index rather than a simple “uptake of screening” rate.

## Conclusions

In summary, our study showed a low level currency of CRC screening among Singaporeans, despite their high level of awareness of the disease. We also found gender differences with Malay females being half as likely as non-Malay females to undergo screening. Other significant gender differences included women being more fearful about endoscopic screening, and being more likely to respond to cues such as public talks and having a family member with CRC. Our findings clearly call for the need to implement gender specific strategies to increase CRC screening. Finally, given the strong association of CRC screening with a doctor’s recommendation for both genders, the influential role of the doctor in promoting screening should be widely promoted.

## Abbreviations

CRC: Colorectal cancer; CT: Computerized tomographic; CI: Confidence interval; FOBT: Fecal occult blood testing; HBM: Heath belief model.

## Competing interests

The authors declare that they have no competing interests.

## Authors’ contributions

RKW conception and design of study, co-ordinated execution of study, interpretation of data, drafted manuscript, WML design of study, execution of study, analysis and interpretation of data, critical appraisal and revision of manuscript, CYH advised on study design statistics, analysis and interpretation of data, ZF execution of study, data acquisition and analysis, CTW conception and design of study, critical appraisal of manuscript, YKG design of study, critical appraisal of manuscript. All authors read and approved the final manuscript.

## Pre-publication history

The pre-publication history for this paper can be accessed here:

http://www.biomedcentral.com/1471-2458/13/677/prepub
